# Structural and Functional Characterization of a Novel Family of Cyclophilins, the AquaCyps

**DOI:** 10.1371/journal.pone.0157070

**Published:** 2016-06-08

**Authors:** Roman P. Jakob, Philipp A. M. Schmidpeter, Johanna R. Koch, Franz X. Schmid, Timm Maier

**Affiliations:** 1 Departement Biozentrum, University of Basel, 4056, Basel, Switzerland; 2 Laboratorium für Biochemie und Bayreuther Zentrum für Molekulare Biowissenschaften, Universität Bayreuth, D-95440, Bayreuth, Germany; Universidad de Granada, SPAIN

## Abstract

Cyclophilins are ubiquitous cis-trans-prolyl isomerases (PPIases) found in all kingdoms of life. Here, we identify a novel family of cyclophilins, termed AquaCyps, which specifically occurs in marine *Alphaproteobacteria*, but not in related terrestric species. In addition to a canonical PPIase domain, AquaCyps contain large extensions and insertions. The crystal structures of two representatives from *Hirschia baltica*, AquaCyp293 and AquaCyp300, reveal the formation of a compact domain, the NIC domain, by the N- and C-terminal extensions together with a central insertion. The NIC domain adopts a novel mixed alpha-helical, beta-sheet fold that is linked to the cyclophilin domain via a conserved disulfide bond. In its overall fold, AquaCyp293 resembles AquaCyp300, but the two proteins utilize distinct sets of active site residues, consistent with differences in their PPIase catalytic properties. While AquaCyp293 is a highly active general PPIase, AquaCyp300 is specific for hydrophobic substrate peptides and exhibits lower overall activity.

## Introduction

Prolyl cis-trans isomerizations are intrinsically slow reactions, which often determine protein-folding reactions [1]. Prolyl isomerases (PPIases) catalyze this interconversion in both peptides and proteins [2]. PPIases are ubiquitous enzymes and three families are known:  the cyclophilins [3], the FK506 binding proteins (FKBPs) [4], and the parvulins [5]. Cyclophilin-type PPIases are involved in a multitude of physiological functions. They act, among others, as chaperones or foldases, for instance in the maturation of steroid hormone receptor complexes [6], in the interplay between NinaA and rhodopsin [7, 8], or are required for the maturation of the HIV capsid protein Gag [9]. Cyclophilins are also critical for cell responses under stress conditions [10, 11] and are involved in the adaptation to environmental stress, in cell cycle control, signal transduction, and transcriptional regulation [11, 12]. In addition, they have been reported to contribute to the virulence of fungal and parasitic pathogens [13–17], and to stress tolerance and pathogenicity of bacteria, such as *Legionella pneumophila* [18], *Enterococcus faecalis* [19], and *Streptococcus pneumoniae* [20].

The prototypic member of the cyclophilin family is human hCyp18 [21–23], a single-domain PPIase of 165 amino acids, with an eight-stranded antiparallel β-barrel core [24, 25]. Most prokaryotic cyclophilins are also monomeric single-domain proteins with variations only in loop regions [26] and are structurally and functionally well characterized [27]. However, some cyclophilins are extended by additional structural elements, such as helical repeat domains, and for most of those extended cyclophilins, structure and function are not known [28, 29].

In the bacterium *Hirschia baltica*, a marine *Hyphomonadaceae* within the class of *Alphaproteobacteria*, we identified two genes that code for novel unusually large cyclophilins. *Hirschia baltica* is suggested to have an important role in marine biofilm formation [30] and has a dimorphic life-cycle; newborn swarmer cells are motile and differentiate into stalked sessile cells, which reproduce by budding motile daughter cells [31]. *Hirschia baltica* contains two cytosolic and five periplasmic canonical single-domain PPIases, but also two large, homologous cyclophilins with 293 and 300 residues, which we term AquaCyp293 and AquaCyp300, respectively. The core PPIase domain of the two AquaCyps shows N- and C-terminal extensions, as well as an insertion, which together double the size relative to single-domain cyclophilins. The structure and function of AquaCyps and their insertion elements are unknown. Single gene knockouts of their respective homologues in *Caulobacter crescentus* had revealed that neither AquaCyp homologue alone is essential in this organism [32].

Here, we determined the crystal structures of both AquaCyp293 and AquaCyp300 and characterized their functional properties. Our data reveal a unique two-domain architecture of AquaCyp proteins, in which the catalytically active cyclophilin domain maintains extensive contacts with a composite domain formed by terminal extensions and internal insertion elements. Both AquaCyps are functional in prolyl isomerase assays but differ with respect to oligomerization, catalytic efficiency and substrate specificity.

## Materials and Methods

### Expression and Purification of AquaCyp293 and AquaCyp300

For the expression of the AquaCyp293 (C6XJ17, res. 25–293) and AquaCyp300 (C6XII3, res. 21–300) the gene fragments were PCR-amplified from *Hirschia baltica* strain ATCC 49814 and cloned into the expression plasmid pNIC28-Bsa4 [33], where they are N-terminally linked to an hexa-histidine tag followed by a TEV-cleavage site. The proteins were overproduced in *E*. *coli* BL21(DE3) *ΔslyD* (gift from B. Eckert). After lysis of the cells in 50 mM Hepes/NaOH, 500 mM NaCl, pH 7.4, 40 mM imidazol with a sonicator and centrifugation, all proteins were found in soluble form. The proteins were purified by immobilized metal-affinity chromatography on a Ni-NTA column (elution with 250 mM imidazole), overnight digested by tobacco etch virus protease [34], followed by a second Ni-NTA chromatography step. The high salt concentrations used during Ni-NTA chromatography significantly reduced the amounts of protein impurities. The flow-through was concentrated in Amicon Ultra units (Millipore) and then subjected to size-exclusion chromatography in 20 mM Hepes/NaOH pH 7.4 on a Superdex S75 column (GE Healthcare). The protein-containing fractions were pooled and concentrated. Yields were about 15 mg L^-1^ culture. RCM-T1 was expressed and purified as described [35].

### Thermal Shift Assay

Solutions of 2 μl protein (100 μM in 20 mM Hepes pH 7.5, 250 mM NaCl, 5% Glycerol) with 8 μl 5× SYPRO Orange (Invitrogen) were added to 10 μl of different buffer solutions to the wells of a 96-well PCR-plate (Biorad). The plates were sealed with highly transparent polypropylene film and heated in an Biorad CFX96 detection system from 4°C to 70°C in increments of 0.5°C. Fluorescence changes in the wells of the plate were measured three times per minute at three different gain settings. The wavelengths for excitation and emission were 492 and 568 nm respectively. The midpoint of unfolding *T*_m_, was derived from the first derivative. The unfolding curves were identical between 0.5 and 5 μM protein. All data were processed using Biorad software.

### Prolyl Isomerase Activity Assay

The prolyl isomerase activities were measured by a protease-free fluorescence assay [36] [37]. For the assay, the peptide substrate Abz-Ala-Xaa-Pro-Phe was dissolved in trifluoroethanol containing 0.5 M LiCl. Under these conditions, about 50% of the peptide molecules are in the cis conformation. The kinetics of the decrease in *cis*-Pro content was measured by the change in fluorescence at 416 nm (5 nm bandwidth) after excitation at 316 nm (3 nm bandwidth) in 100 mM K-phosphate 7.0 at 15°C. Under these conditions, the cis-to-trans isomerization of the prolyl bond was a mono-exponential process, and its rate constant was determined by using GraFit 3.0 (Erithacus Software, Staines, UK). The folding experiments of RCM-T1 were performed as described [38] (Table 1).

**Table 1:** Catalytic efficiencies of AquaCyp293 and AquaCyp300 for prolyl isomerization in peptide and protein substrates.

	AquaCyp293 ^a^	AquaCyp300 ^a^	hCyp18 ^b^
*Peptide isomerization*	*k*_cat_/*K*_M_ (mM^-1^s^-1^)	*k*_cat_/*K*_M_ (mM^-1^s^-1^)	*k*_cat_/*K*_M_ (mM^-1^s^-1^)
Abz-Ala-**Ala**-Pro-Phe-pNA	4800 ±4.1	63 ±2	8690 ± 280
Abz-Ala-**Glu**-Pro-Phe-pNA	1100 ±13	23 ±3.9	9870 ± 30
Abz-Ala-**Leu**-Pro-Phe-pNA	7700 ±500	1500 ±6.6	6910 ± 410
Abz-Ala-**Lys**-Pro-Phe-pNA	1700 ±39.5	14 ±2.6	4020 ± 20
Abz-Ala-**Phe**-Pro-Phe-pNA	2400 ±200	200 ±3.9	4720 ± 40
*Protein folding*			
RCM-T1 ^c^	10 ±0.24	2 ±0.34	38 ± 5

^*a*^ Catalytic activities towards peptides from protease-free experiments as described in the legend to Fig 3. The confidence limits of the *k*_cat_/*K*_*m*_ values were between 5 and 10%.

^*b*^ Catalytic activity data towards peptides are taken from Ref. 37.

^*c*^ Catalytic activity was determined using the refolding of RCM-T1 in 2 M NaCl, 100 mM Tris-HCl, pH 7.8, and 1 mM EDTA.

### Chaperone Activity Assay

Citrate synthase was unfolded in 50 mM Tris–HCl (pH 8.0), 20 mM dithioerythritol, 6 M GdmCl for 1 h and then diluted 200 fold to a final concentration of 0.15 μM (monomer) in 50 mM Tris–HCl (pH 8.0), 0.1 mM dithioerythritol, 30 mM GdmCl, and various concentrations of PPIase at 25°C. Spontaneous aggregation was monitored by measuring the increase in light scattering at 360 nm.

### Size Exclusion Chromatography Coupled with Multi-Angle Light Scattering

For size exclusion chromatography coupled with multi-angle light scattering (SEC-MALS) measurements, 20- or 100-μl samples of 1 mg/ml of AquaCyp293 or AquaCyp300 were applied to a GE Healthcare Superdex 200 5/150 GL SEC column equilibrated overnight in 20 mM Hepes, pH 7.4, at 5°C, using an Agilent 1100 series HPLC system. Light scattering and differential refractive index measurements were made using a Wyatt miniDawn TriStar detector and a Wyatt Optilab rRex detector, respectively. The inter-detector delay volumes, band broadening, and the light scattering detector normalization, were calibrated according to the manufacturer's protocol using a 2 mg/ml of BSA solution (Thermo Pierce) in the same buffer. The absolute refractive index of the buffer was measured using the refractive index detector. The data were collected and processed using Wyatt Astra 5 software. The molar mass was calculated from a global fit of the light scattering signals from three detectors at different angles, and the differential refractive index signal, using algorithms in the Astra 5 software.

### Protein Crystallization and Structure Determination

AquaCyp293 and AquaCyp300 were crystallized by vapor diffusion using the sitting-drop method at 20°C. The reservoir solution (100 μl) contained 10% PEG20000, 20% PEGMME550 in 0.03 M CaCl_2_, 0.03M MgCl_2_, 0.1 M Mops/Hepes-Na pH 7.5. Diffraction data were collected at the Swiss Light Source PXI beamline. The data sets were processed and scaled using XDS [39, 40]. The AquaCyp293 crystals belong to space group P_1_2_1_1 with cell dimensions *a* = 47.9 Å, *b* = 72.7 Å, and *c* = 73.9 Å, β = 93° and contain two molecules per asymmetric unit. AquaCyp300 also crystallized in space group P_1_2_1_1 with cell dimensions *a* = 50.6 Å, *b* = 103.2 Å, and *c* = 173.5 Å, β = 91.5° containing six molecules in the asymmetric unit. Structure determination of AquaCyp293 was performed by molecular replacement with the human cyclophilin A structure as the search model (Protein Data Bank accession code 2CPL) using Phaser [41]. Model building and structure refinement were performed with Coot [42] and PHENIX [43], respectively (Table 2). The atomic coordinates for AquaCyp293 and AquaCyp300 have been deposited in the RCSB Protein Data Bank and are available under the accession code 5EX2 and 5EX1, respectively.

**Table 2:** Statistics on diffraction data and structure refinement of the AquaCyp293 and AquaCyp300.

Data set	AquaCyp293	AquaCyp300
Space group	P_1_2_1_1	P_1_2_1_1
Unit cell	47.9 72.7 73.9 90 93 90	50.6 103.2 173.5 90 91.2 90
Resolution (Å)	47.8–1.30 (1.37–1.3) ^a^	88.6–2.05 (2.18–2.05) ^a^
Total reflections	762480	731570
Unique reflections	122364	110412
Multiplicity	6.2 (6.1)	6.6 (6.8)
Completeness (%)	97.1 (90.2)	99.2 (92.7)
Mean I/sigma(I)	8.0(1.1)	12.4(2.4)
Wilson B-factor	11.38	26.59
R-merge	0.117 (1.6)	0.166 (1.049)
CC_1/2_	99.6 (56.6)	99.6 (72.2)
R-work	0.1698 (0.3236)	0.1763 (0.2380)
R-free	0.1956 (0.3361)	0.2132 (0.2705)
Number of atoms	9646	27083
macromolecules	4321	12910
ligands	4	3
water	1098	1511
Protein residues	533	1630
RMS(bonds)	0.015	0.006
RMS(angles)	1.56	1.02
Ramachandran favored (%)	98	97
Ramachandran outliers (%)	0	0.19
Clashscore	2.4	1.6
Average B-factor	16.4	35.1
macromolecules	13.5	34.7
ligands	13.8	28.3
solvent	27.9	39

^*a*^ Values in parentheses are for highest resolution shell.

## Results and Discussion

### AquaCyps, a Novel Class of Cyclophilins Characteristic of Marine Alphaproteobacteria

**Fig 1:**
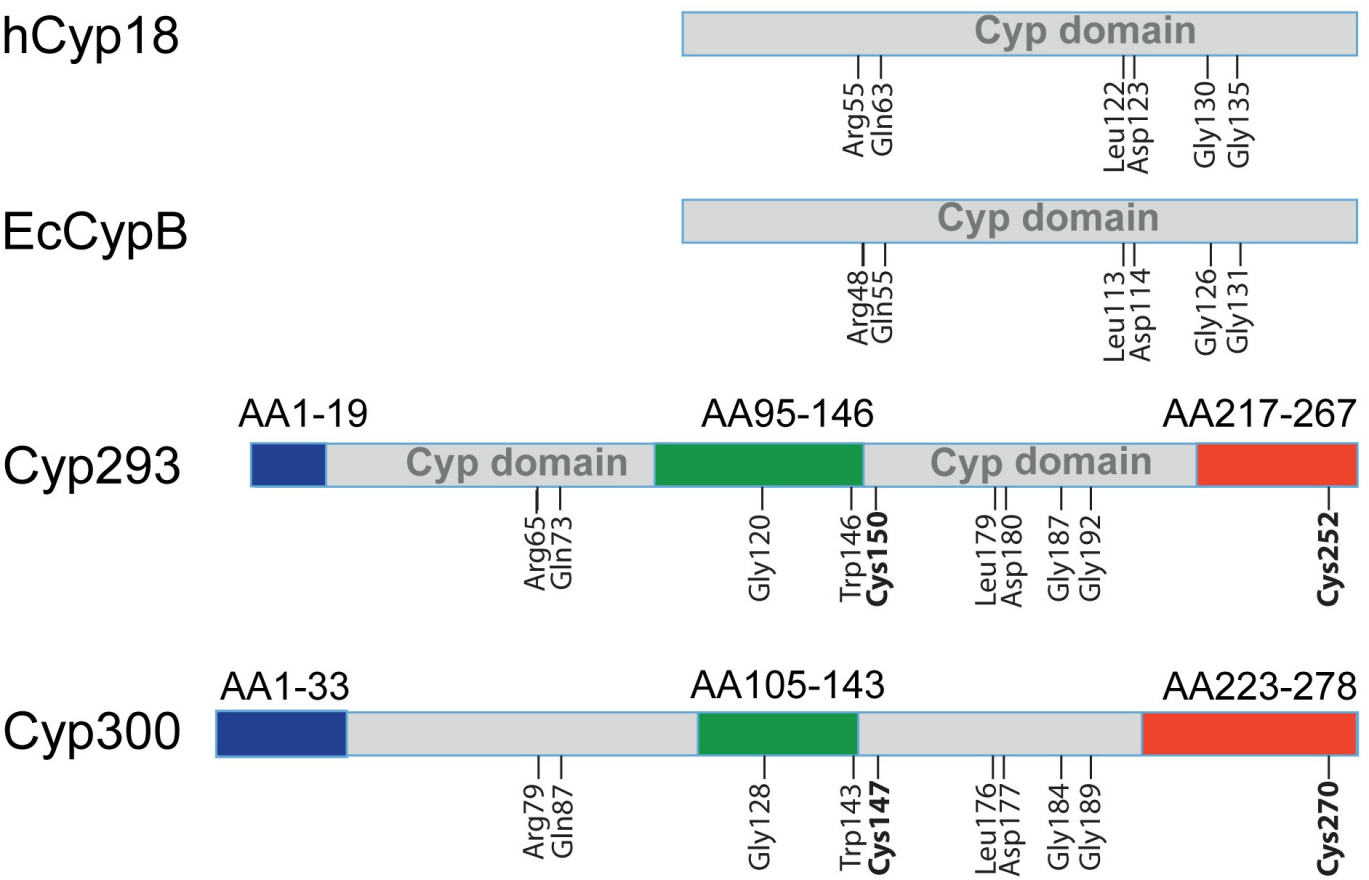
Domain structure and conservation of AquaCyp. The cyclophilin domains are shown in grey, the additonal N-terminal-, insertion, and C-terminal structural elements are colored in blue, green and red, respectively. The indicated amino acids are conserved among cyclophilins. The two invariant cysteine residues in AquaCyp are shown in bold. Extension and insertion regions are indicated above the sequences.

To identify cyclophilins that possibly assist in the maturation of periplasmic and outer membrane proteins [44], we searched the Expasy database [45] for homologs of EcCypB (PPIB), the periplasmic cyclophilin of *E*. *coli*. We found that EcCypB is not generally conserved in *Alphaproteobacteria*, but several of them, such as *Rhodobacterales*, *Rhizobiales* and *Caulobacterales*, contain larger EcCypB homologues of 280 to 330 residues. We found about 50–60 orthologues of these cyclophilins, and because they are almost exclusively present in organisms living in marine environments, we named this cyclophilin class AquaCyps. Interestingly, *Hirschia baltica* contains two such cyclophilins, AquaCyp293 and AquaCyp300, which exhibit 39% sequence identity. All members of this family contain large N-terminal (20–30 residues) and C-terminal extensions (50–60 residues) relative to hCyp18 as well as a 40–50 residue insertion in the long loop that connects the β strands 4 and 5 of the cyclophilin domain (S1 Fig). Members of this family also show two conserved cysteine residues (Cys150 and Cys252 in AquaCyp293) that might form a disulfide bond between the Cyp domain and the C-terminal extension (Fig 1).

The cyclophilin domain of AquaCyp contains several invariant residues that are conserved in all cyclophilins. These include Leu179 and Asp180 (Leu113 and Asp114 in hCyp18), which are part of the characteristic 3_10_ helix of the cyclophilin fold and the two glycine residues 186 and 191 (Gly126 and Gly131 in hCyp18), which are located in sterically demanding positions (Fig 1). Active site residues in AquaCyp are only partially conserved to other cyclophilins. The residues Arg65 and Gln73 of AquaCyp293 correspond to Arg55 and Gln63 in hCyp18 and are essential for catalysis [46–48]. At other positions in the active site, the sequence conservation is lower.

### AquaCyps Exhibit a Low Thermal Stability

**Fig 2:**
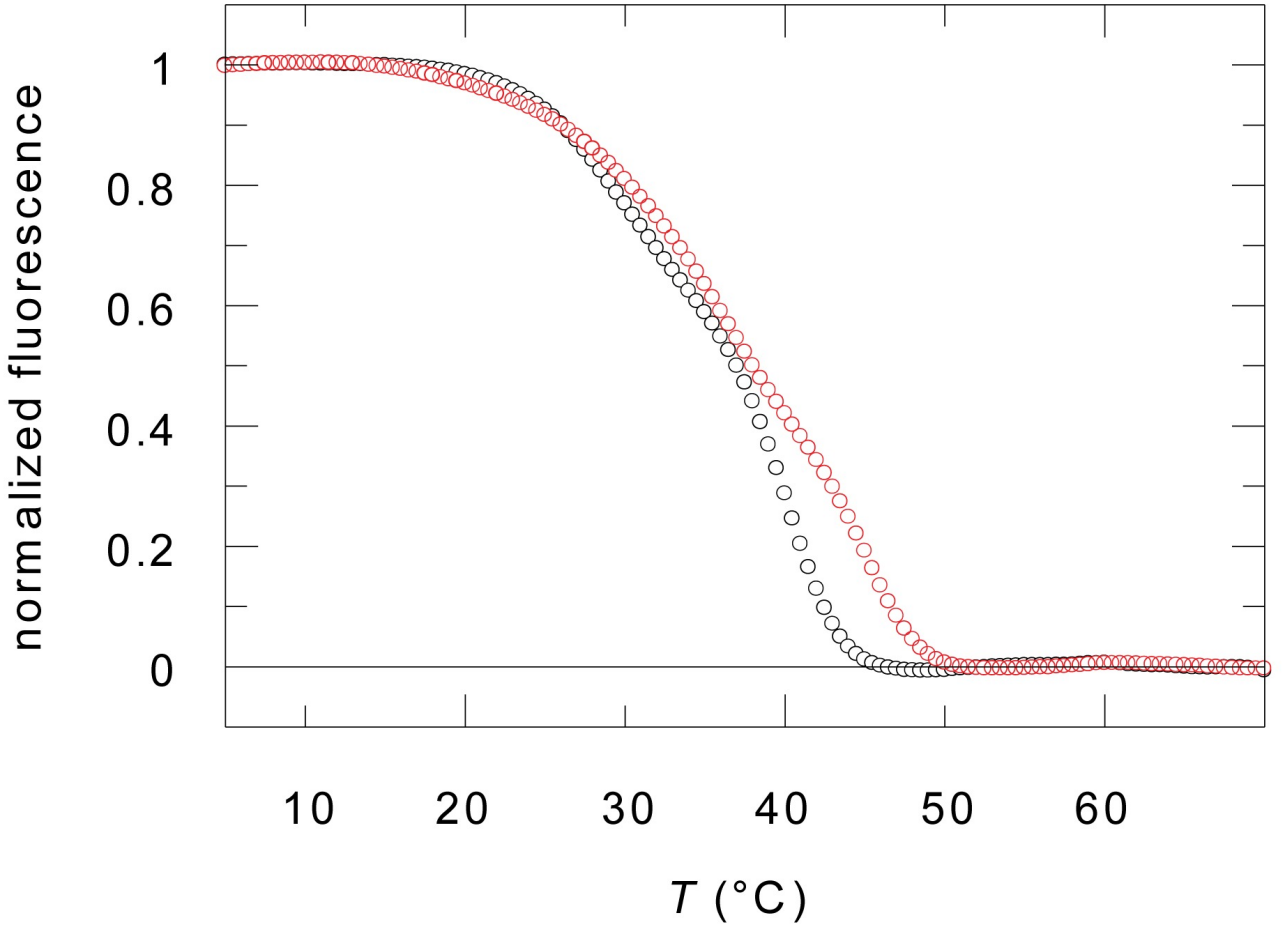
Thermal unfolding of AquaCyp293 (red) and AquaCyp300 (black). Unfolding was followed by the fluorescence change of SYPRO orange at 570 nm after excitation at 490 nm and measured with 2 μM protein in 50 mM Hepes 7.5, 250 mM NaCl, 100 mM KOAc. The unfolding curves were identical between 0.5 and 5 μM protein.

We produced recombinant versions of full length AquaCyp293 and AquaCyp300 and applied differential scanning fluorometry screening to optimize buffer conditions for protein stability. Based on these fluorescence-based thermal stability assays [49], both proteins were found to be folded at room temperature and between pH 6 and pH 8. AquaCyp293 and AquaCyp300 unfolded in several steps resulting in broad melting curves with midpoints at about 38–40°C (Fig 2). These melting points are about 5–10°C lower than those of other prokaryotic and eukaryotic cyclophilins [50, 51]. The lower stability might be correlated to the temperature growth optimum of *Hirschia baltica* in seawater at 22–28°C [52]. Optimal stability was observed in 20 mM Hepes pH 7.4, containing 250 mM NaCl and 10% glycerol. This buffer was subsequently used for protein storage. Its pH is significantly higher than the pI values of 4.7 and 4.8, calculated for AquaCyp293 and AquaCyp300, respectively. AquaCyp293 is a monomeric protein based on size exclusion chromatography—multi angle light scattering (SEC-MALS) (S2 Fig). However, AquaCyp300 elutes in SEC-MALS as a mixture of monomers (60%) and dimers (40%) when injected at a protein concentration of 1 mg/ml. The two separated SEC-MALS elution peaks of AquaCyp300 suggest a slow monomer/dimer transition (S2 Fig).

### AquaCyps Do Not Display Molecular Chaperone Activity

Cyclophilin proteins from several organisms show molecular chaperone activity [53–55]. To examine whether AquaCyp293 and AquaCyp300 interact with unfolded proteins and prevent aggregation, we employed the widely used citrate synthase (CS) aggregation and inactivation assay [56]. It exploits the strong aggregation tendency of folding intermediates of citrate synthase. Unfolded citrate synthase aggregates spontaneously after dilution with refolding buffer, which is accompanied by a strong increase in light scattering. AquaCyp293 and AquaCyp300 were both unable to suppress the temperature-induced aggregation of CS even when added in large excess (S3 Fig). In a control experiment, the chaperone SlyD from *E*. *coli* abolished aggregation under the same conditions [57].

### Only AquaCyp293 Is an Efficient General PPIase

Most prolyl isomerases of the cyclophilin family catalyze prolyl isomerization in peptides and proteins with very high efficiency but show low sequence specificity for the amino acid preceding proline [58, 59]. To examine the catalytic activity of AquaCyp293 and AquaCyp300, we used a fluorimetric protease-free assay and proline-containing tetrapeptides that carry an aminobenzoyl (Abz) group at the aminoterminus and a para-nitroanilide (pNA) group at the carboxyterminus. The short peptides have the general formula Abz-Ala-Xaa-Pro-Phe-pNA. In these peptides, the Xaa position was occupied by a charged (Glu, Lys), an aliphatic (Ala, Leu), or an aromatic (Phe) residue.

**Fig 3:**
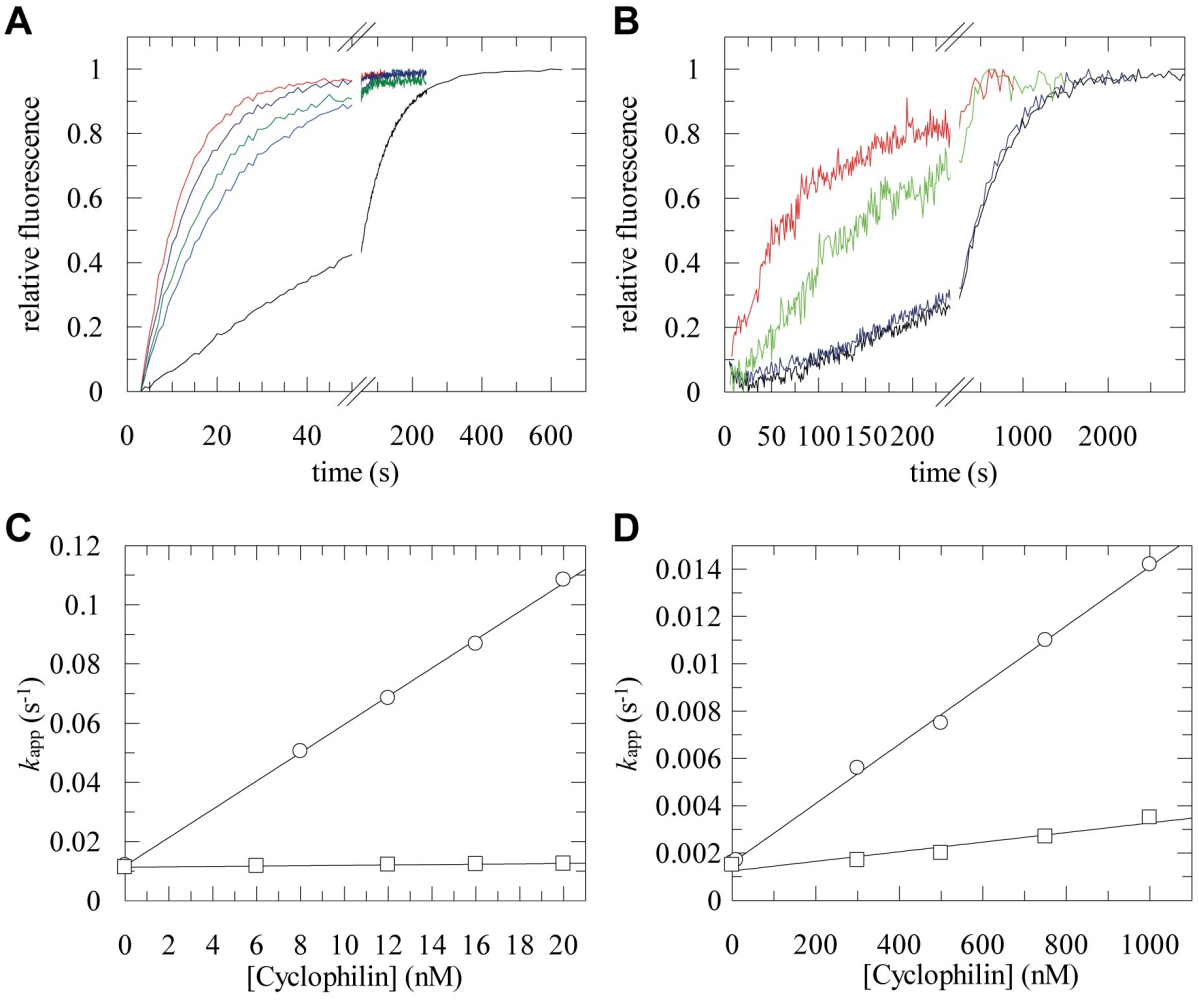
PPIase activities of AquaCyp293 and AquaCyp300. (A) Kinetics of *cis*/*trans* isomerization of 3 μM Abz-Ala-Ala-Pro-Phe-pNa followed by fluorescence at 416 nm, without enzyme (black), with 8 nM (blue), 12 nM (green), 16 nM (dark blue) and 20 nM (red) AquaCyp293. (B) Refolding kinetics of RCM-T1 in the presence of increasing concentrations of AquaCyp293, 0 nM (black), 10 nM (blue), 300 nM (green) and 750 nM (red). The kinetics of refolding of 0.1 μM RCM-T1 in 0.1 M Tris/HCl pH 8.0; 2 M NaCl were measured at 15°C in the presence of various concentrations of AquaCyp293. (C, D) Catalytic efficiencies of AquaCyp293 (○) and AquaCyp300 (□) for (C) the *cis*/*trans* isomerization of Abz-Ala-Ala-Pro-Phe-*p*NA and (D) the refolding of RCM-T1. The measured proline-limited refolding rate constants *k*_app_ are shown as a function of the PPIase concentration. The *k*_cat_/*K*_m_ values derived from the slopes are given in Table 1.

AquaCyp293 is a highly active PPIase (Fig 3A–3C). 12 nM AquaCyp293 accelerated the isomerization of Abz-Ala-Ala-Pro-Phe-pNA 5-fold (Fig 3A). From measurements of the isomerization rate as a function of the AquaCyp293 concentration (as in Fig 3C), the catalytic efficiencies (*k*_cat_/*K*_M_) for five Xaa-Pro sequences were derived (Table 1). Similar to the highly active human Cyp18, AquaCyp293 shows very high *k*_cat_/K_M_ values, which range between 10^6^ and 10^7^ M^-1^s^-1^, and the maximal differences between peptides are only 6-fold (between Leu-Pro and Glu-Pro). AquaCyp293 thus resembles other cyclophilins in both high PPIase activity and low substrate specificity [37, 58, 60].

AquaCyp300 is active as a PPIase as well (Table 1), but it differs significantly from AquaCyp293 and human Cyp18. For four out of the five peptides tested, the activities are 12-120-fold lowered relative to AquaCyp293. A moderate PPIase activity was observed only for the Leu-Pro containing peptide, pointing to pronounced substrate specificity for hydrophobic residues preceding proline. To our knowledge, such high substrate specificity has hitherto been observed only for prolyl isomerases of the FKBP family [36], not for cyclophilins [37]. The differences in activity and substrate specificity suggest that AquaCyp293 and AquaCyp300 might have distinct functions in the periplasm of *Hirschia baltica*.

Next, we investigated the efficiencies of the two AquaCyp enzymes in the catalysis of a proline-limited protein folding reaction. Reduced and carboxymethylated RNase T1 (RCM-T1) was used as the model substrate (Fig 3B). Its refolding reaction is limited in rate by the *trans*→*cis* isomerization of a Tyr-Pro bond. Refolding can be induced by increasing the NaCl concentration [61, 62] and is monitored by the strong increase in tryptophan fluorescence upon refolding. AquaCyp293 and AquaCyp300 contain four tryptophan residues and thus contribute strongly to the measured fluorescence, which decreases the signal/noise ratio in the folding assays (Fig 3B). With a *k*_cat_/*K*_M_ value of 1.0 × 10^4^ M^-1^ s^-1^, AquaCyp293 catalyzes prolyl isomerization in a folding protein about 100-fold less efficiently than in the tetrapeptide substrates. With these catalytic properties it resembles human Cyp18, as well as CypA from *Escherichia coli* and *Bacillus subtilis* [58, 60]. AquaCyp300 also catalyzes RCM-T1 refolding, but is about fivefold less efficient than AquaCyp293 (Fig 3D).

### AquaCyps Share Characteristic Extensions of the Cyclophilin Fold

AquaCyp293 and AquaCyp300 were crystallized under similar conditions. Plate like crystals appeared in mixtures of short and long polyethylene glycols at neutral pH in the presence of divalent cations. The X-ray crystal structures of AquaCyp293 and AquaCyp300 were solved by molecular replacement with the structure of hCyp18 (pdb: 2CPL) as a search model and refined at resolutions of 1.3 Å and 2.05 Å to *R*_*work*_/*R*_free_ values of 17.0/19.5% and 17.6/21.3%, respectively. Data collection and refinement statistics are shown in Table 2. The crystallographic asymmetric units for AquaCyp293 and for AquaCyp300 contain two and six molecules, respectively. The individual AquaCyp293 and AquaCyp300 molecules are virtually identical at root mean square deviation of mainchain atom positions (rmsd) of 0.1 and 0.2 Å, respectively (S4A and S4B Fig). Minor differences in surface exposed loops probably originate from crystal packing. In both crystal structures Mg^2+^ ions mediate crystal contacts.

**Fig 4:**
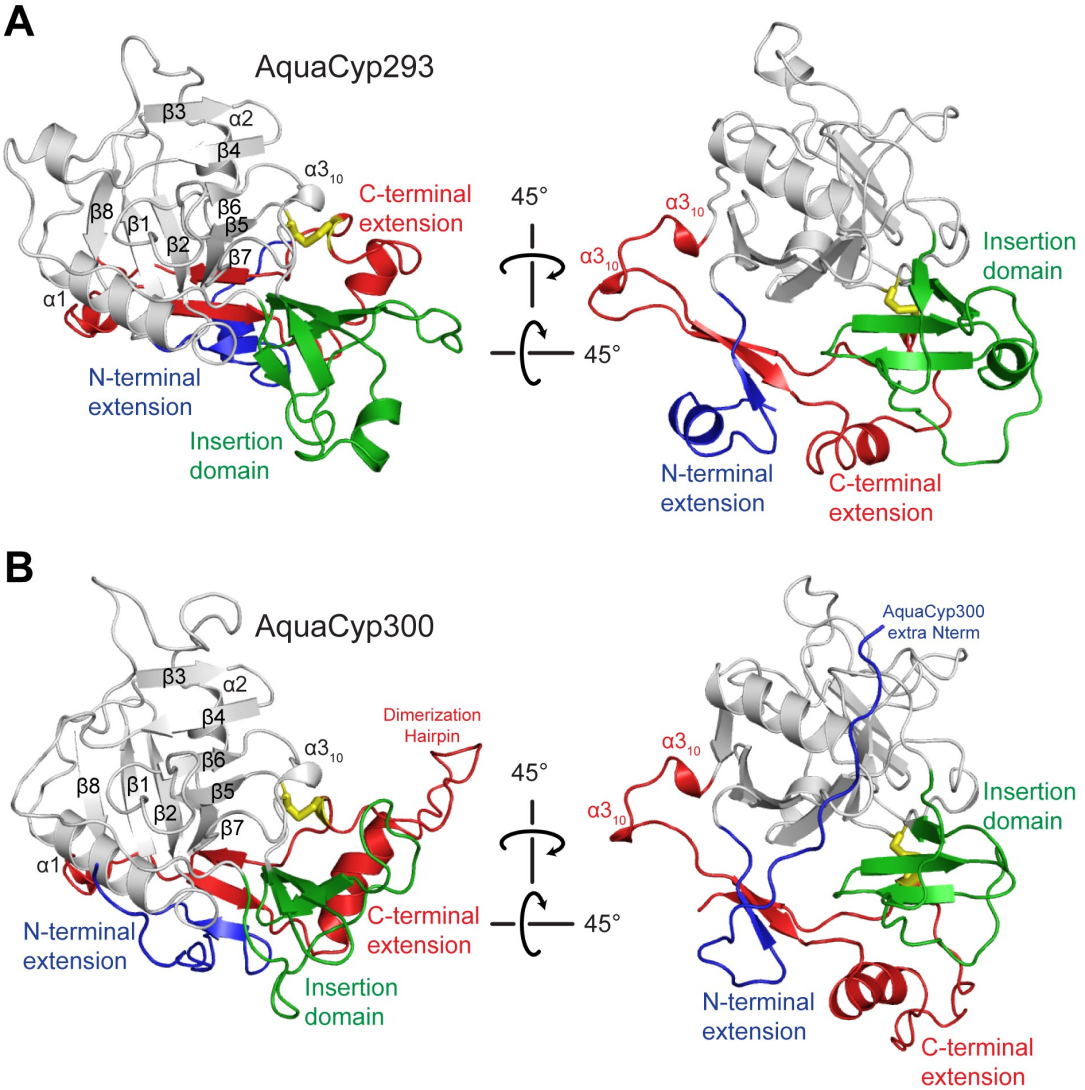
Crystal structures of AquaCyp293 and AquaCyp300. Domain architecture and topology of AquaCyp293 (A) and AquaCyp300 (B). The cyclophilin fold in grey consists of an eight stranded-antiparallel β-barrel and two α-helices covering the top and the bottom of the barrel. The disulfide bridge (yellow) is shown in stick representation. The additional N-terminal-, insertion, and C-terminal structural elements are coloroured in blue, green and red, respectively.

The cyclophilin domains of both AquaCyp293 and AquaCyp300 consist of an eight-stranded antiparallel β-barrel, and two *α*-helices covering the top and the bottom of the barrel, and an additional small 3_10_-helix (Fig 4A and 4B). They superimpose well with hCyp18 with rmsd values of 0.9 Å and 1.0 Å, respectively (S4C and S4D Fig). The closest homologue for both, AquaCyp293 and AquaCyp300, is EcCypB from *E*. *coli* [63], which also is a periplasmic PPIase. All secondary structure elements of the AquaCyp293 and AquaCyp300 cyclophilin domain superpose well with EcCypB, differences are restricted to loop regions (S4E and S4F Fig).

29 of the 30 cyclophilin structures in the protein data bank are single domain proteins (155–186 residues). Bovine Cyp40 is the only structurally resolved cyclophilin with an extra domain, which, in this case is a C-terminal tetratricopeptide repeat (TPR) domain [64]. All single-domain cyclophilin structures superpose well with an rmsd of better than 1.5 Å onto the cyclophilin domain of AquaCyp293 and AquaCyp300 showing that the extra sequence regions in AquaCyps virtually do not affect the structure of the cyclophilin domain. A phylogenetic analysis of cyclophilins with known structures revealed that AquaCyp293 and AquaCyp300 are both located together in an independent branch in the phylogenetic tree of cyclophilins (S5 Fig) demonstrating their close similarity.

### Variations in Active Site Structure between AquaCyp293 and AquaCyp300

**Fig 5:**
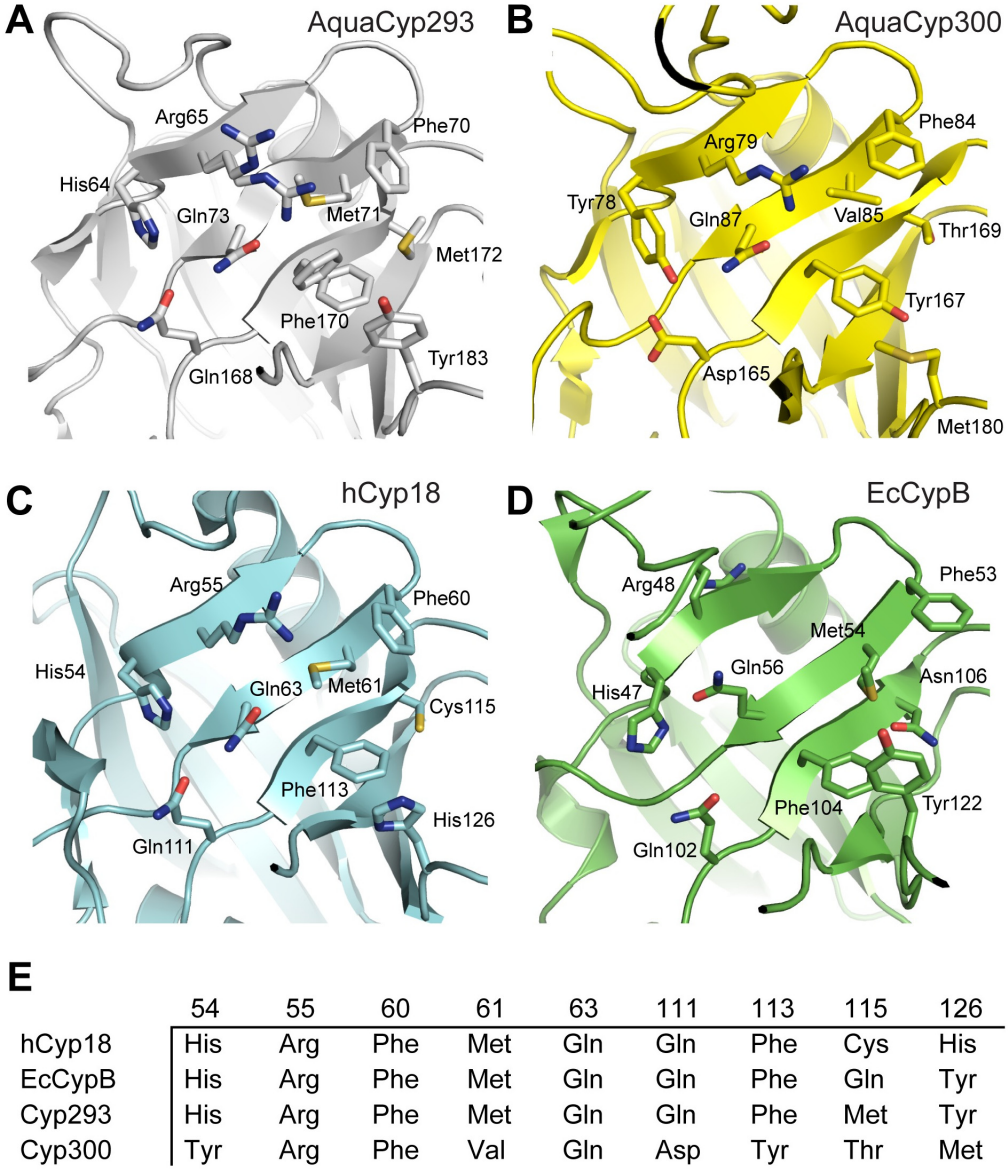
Active site structure of AquaCyp293 (A) and AquaCyp300 (B) in comparison to hCyp18 (C) and EcCypB (D). Residues that contribute to the active site of the cyclophilin family are labeled and shown in stick representation. (E) Conservation of PPIase active site residues. Residue numbering according to hCyp18 (C).

Residues forming the active site of cylcophilins are mainly located on β-strands 3, 4 and 6 [65]. Most of the residues known to be important for activity in hCyp18 and EcCypB are also conserved in AquaCyp293 (Fig 5), in agreement with their catalytic competence (Table 1). In the high-resolution structure of AquaCyp293, alternative side chain conformations are observed for Arg55 and Phe113 (Fig 5A), suggesting that flexibility is important for substrate binding and catalysis. Although many high-resolution cyclophilin structures are known, such alternate conformations for active site residues have rarely been reported for cyclophilins. However, the relevance of alternate conformations of active site residues in cyclophilins for catalysis was demonstrated by NMR and ambient-temperature X-ray crystallographic data collection for human hCyp18 [66].

In contrast to AquaCyp293, AquaCyp300 (Fig 5B) differs from hCyp18 (Fig 5C) and EcCypB (Fig 5D) at six out of nine active site positions (Fig 5E). Only Arg55, Phe60 and Gln63, which are most important for catalysis [65], are conserved. The residues His54, Gln111, His126, which contribute to the PPIase activity of hCyp18 as well, are substituted by Tyr, Asp, Met, respectively (Fig 5E) [67], providing a plausible explanation for the low PPIase activity and high substrate specificity of AquaCyp300.

### Core-Fold Extensions in AquaCyps Form a Contiguous Structural Entity, the NIC Domain

The three extra chain regions of AquaCyp293 and AquaCyp300 outside the PPIase domain are well ordered. Although the terminal extensions and the insert are separated in sequence space, they form a contiguous structural entity in both proteins, which we term the NIC domain (Fig 4).

In AquaCyp293, the N-terminal extension starts with a short α-helix (Lys5-Asp11), which is followed by a short β-strand (Asn14-Val18) (blue in Fig 4A) that pairs with a β-strand (Pro226-Met231) provided by the C-terminal extension (red in Fig 4A). The insert in the cyclophilin domain (residues 95–146, green in Fig 4A) folds into a four-stranded (parallel-antiparallel) β-sheet (Val95-Asp99; Ser116-Tyr122; Phe125-Arg130; Arg142-Met147) and an α-helix (Glu131-Thr137). The C-terminal extension forms an elongated structure that contacts the N-terminal extension, the inserted domain, and the cyclophilin (PPIase) domain. It starts with two short 3_10_ helices and continues into β-strands, which provide a structural link with the β-strand of the N-terminal extension and a β-strand (Ser259-Phe262) of the cyclophilin domain.

In AquaCyp300, the first eight residues are not resolved in the crystal structure, presumably because they are mobile. Ile9 is ordered and packs against Asn160 in the loop between β-strands 5 and 6 of the cyclophilin domain (Fig 4B). The subsequent residues of the N-terminal extension (blue in Fig 4B) wrap around the cyclophilin domain forming a loop that packs against the C-terminal residues, then going back and in a short β-strand (Gly26-Ile32) leading into the cyclophilin domain. As for AquaCyp293, the large insertion in the cyclophilin domain is located in the long loop between β-strand 4 and 5 and consists of loops and three short β-strands (Lys126-Leu130; Phe133-Asp138; Glu140-Leu145) (green in Fig 4B). It is placed between the N-terminal and C-terminal extensions and overlaps well with the insertion domain of AquaCyp293. The C-terminal extension is the largest additional part of AquaCyp300 (red in Fig 4B). After the cyclophilin domain, it starts with two short 3_10_ helices and then it forms a β-strand (Asn233-Met237) that pairs on one site with the β-sheet from the N-terminal extension and on the other with the β-strand (Pro275-Val279). This β-strand is followed by a α-helix (Ala242-Arg252), which leads into a long hairpin. The NIC domains of AquaCyp293 and AquaCyp300 superimpose well with an rmsd of 1.5 Å and apparently represent novel domain folds. A structural similarity search with DALI did not reveal structural homologs with a Z-Score above 2 [68].

### A Disulfide Bond Tethers the NIC to the Cyclophilin Domain in AquaCyps

Both AquaCyp293 and AquaCyp300 possess a buried disulfide bond at structurally equivalent positions (Fig 4A and 4B). It is formed by a cysteine residue in the cyclophilin domain (Cys150 in AquaCyp293; Cys147 in AquaCyp300) and a cysteine in the C-terminal extension (Cys252 in AquaCyp293; Cys272 in AquaCyp300) and firmly links the C-terminal extension to the cyclophilin domain. The disulfide bond is remote from the active site, and we suggest that it has a structural rather than a functional role. In two other reported cases, disulfide bonds in cyclophilins are linked to enzymatic activity: In CypA from *Schistoma masoni*, SmCypA, the disulfide bond is solvent exposed and located close to the active site. A regulation mechanism via oxidation has been suggested, as the oxidized form is inactive, whereas the reduced form of SmCypA shows high PPIase activity [69]. CYP20-3 from *Arabidopsis thaliana* also contains a disulfide bond close to the active site, and its enzymatic activity is decreased in the oxidized form [70].

### The NIC Domain Mediates Dimerization of AquaCyp300

**Fig 6:**
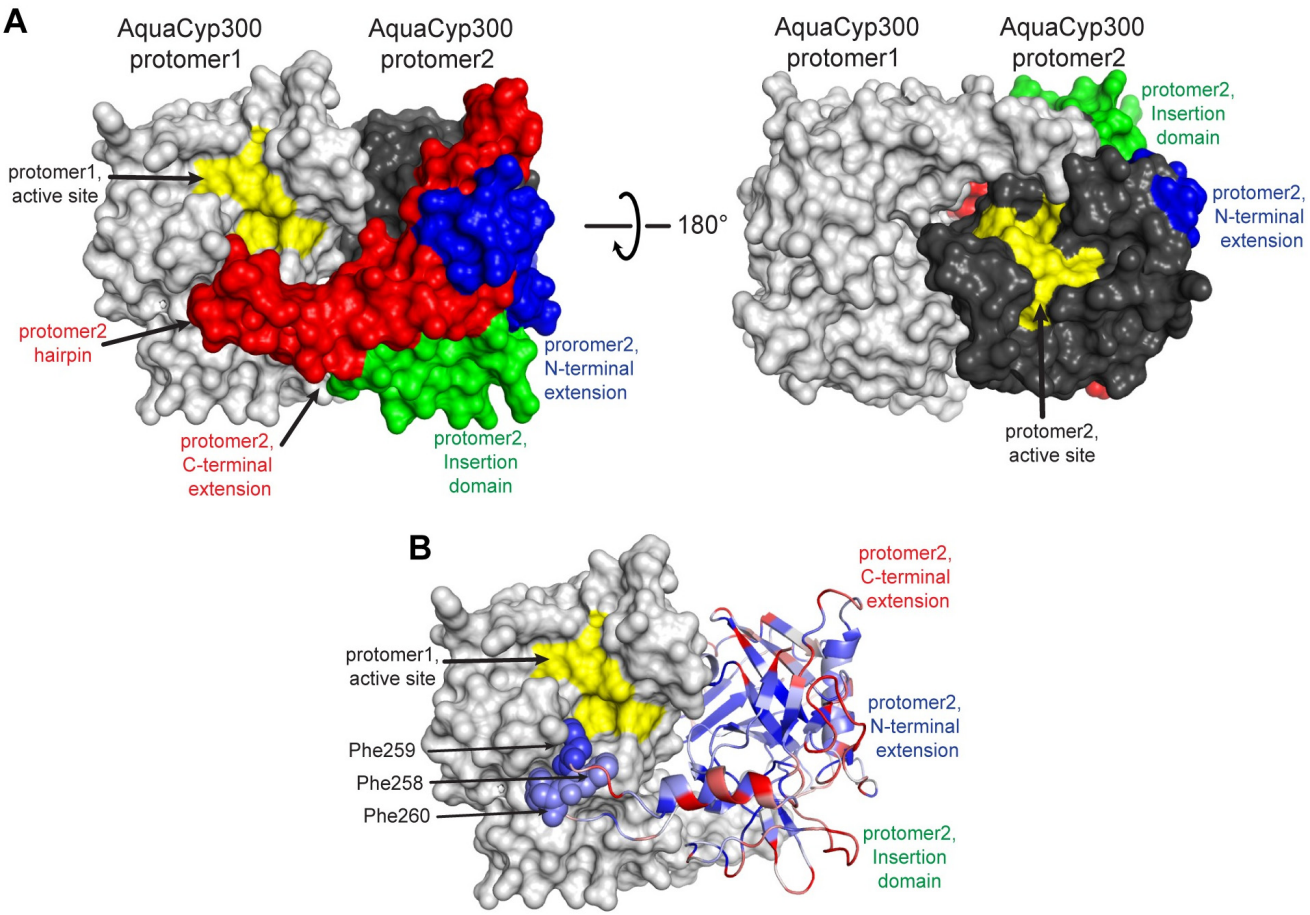
Dimerization of AquaCyp300. (A) Surface representation of the dimeric AquaCyp300 crystal structure, one protomer is colored in light the other in dark grey, the N-terminal-, insertion, and C-terminal structural elements in one protomer are colored blue, green and red, respectively, active site residues of both protomers are colored yellow. (B) Sequence conservation [83] within the AquaCyp300 family is mapped onto a cartoon representation of AquaCyp300 protomer2. Residues that are highly conserved (e.g. Phe258, Phe259, Phe260) are shown in blue, sequences with lower identity are shown in white and red.

The SEC-MALS analysis demonstrated that AquaCyp300 in solution is in monomer/dimer equilibrium. In the AquaCyp300 crystal structure the six monomers are indeed arranged into three dimers. 50 residues form the AquaCyp300 dimer with an interface area of 1713.4 Å^2^ (12.9% of the molecule surface) [71], placing the active sites of AquaCyp300 on opposing faces of the dimer (Fig 6A). Residues from both the cyclophilin domain and the NIC domain contribute to dimerization. They show low conformational flexibility (S6 Fig) and high sequence conservation (Fig 6B), suggesting that other AquaCyp300 homologues are also dimeric. In particular, an extended hairpin structure enriched in hydrophobic residues (S7 Fig) (including three phenylalanines Phe258-Phe260) at the C-terminal extension interlocks the two monomers (Fig 6C). The tip of this dimerization hairpin is in close proximity to the respective active site of the related dimeric AquaCyp molecule and might be involved in substrate interaction. To our knowledge AquaCyp300 is the first reported dimeric cyclophilin. For FKBP or parvulin containing PPIases several dimeric PPIases (e.g. FkpA, Fkbp22, Mip, PrsA and Peb4) are known, where extra alpha-helical domains mediate dimerization and in addition function as chaperones. In these dimers, the PPIase domains are not on opposite sides but face each other, and this combination of chaperone and prolyl isomerase activity is thought to be important for an optimal function as protein folding enzyme [44, 72–76].

## Conclusion

Protein maturation in the periplasm is a complex process that involves a network of foldases and chaperones. In a bioinformatics analysis, we identified a distinct class of cyclophilin PPIases with complex architecture in organisms with a dimorphic, marine lifestyle, such as *Hyphomonas*, *Caulobacter* and *Hirschia*. To our knowledge, it represents the first class of periplasmic cyclophilins with additional non-catalytic domains. The crystal structures of AquaCyp293 and AquaCyp300 demonstrate that AquaCyps fold into two domains, a classical cyclophilin-type PPIase domain and the NIC domain with a novel mixed alpha-helical/beta-strand structure. Both AquaCyp proteins are stabilized by a conserved inter-domain disulfide bond. Despite pronounced structural similarities, they also show distinct features: AquaCyp293 is monomeric and resembles canonical cyclophilins in its enzymatic properties, whereas AquaCyp300 dimerizes via the NIC domain at higher protein concentration and shows low enzymatic activity and high substrate specificity, suggesting distinct functions for the two PPIases. Indeed, the vast majority of marine *Alphaproteobacteria* contain both AquaCyp homologous. Marine Organisms with dimorphic life style encode a large number of characteristic proteins that are components of the outer membrane or involved in cell envelope biogenesis [30, 52, 77]. Presumably, AquaCyps are involved in the maturation of one or more of these proteins. Their distinct catalytic properties suggest distinct sets of target proteins, but don’t exclude partially overlapping functions. Further analysis will be required to identify the substrates of the two AquaCyp PPIases and to reveal their interplay with other folding helpers in the periplasm of marine Alphaproteobacteria.

## Supporting Information

S1 Fig Domain structure and sequence conservation of AquaCyp. Multiple sequence alignment to analyze the sequence and secondary structure conservation of PrsA. Highly conserved residues are red (>70% conservation) or white in red boxes (100% conservation). On top, the cyclophilin domain are shown in grey, the additonal N-terminal-, insertion, and C-terminal structural elements are coloroured in blue, green and red, respectively. The secondary structure of *Homo sapiens* hCyp18 is shown on top of the protein sequence. Sequences of representative AquaCyp proteins were retrieved from the UniProt database [78] and aligned using MULTALIN [79]. The final figure was generated using the ESPript server [80]. Species abbreviations and UniProt accession numbers are AquaCyp293, *Hirschia baltica* (C6XJ17); HpAquaCyp, *Hyphomonas polymorpha PS728* (A0A062V843); HsAquaCyp, *Hyphomonas johnsonii MHS-2* (A0A059F982); AsAquaCyp, *Asticcacaulis sp*. *AC460* (V4PTX4); AbAquaCyp, *Asticcacaulis biprosthecum C19* (F4QR87); PzAquaCyp, *Phenylobacterium zucineum (strain HLK1)* (B4R9P8); AquaCyp300, *Hirschia baltica* (C6XII3); CpAquaCyp, *Colwellia psychrerythraea (Vibrio psychroerythus)* (A0A099L3T0); PpAquaCyp, *Paraglaciecola polaris LMG 21857* (K6ZRP5); Q1AquaCyp, *alpha proteobacterium Q-1* (A0A061QDA5).
(TIF)

S2 Fig Molecular weight determination using size-exclusion chromatography coupled to static light scattering. The chromatograms are shown for AquaCyp293 (red) and AquaCyp300 (blue). The molecular mass was calculated throughout the eluting peaks and is indicated in red (AquaCyp293) and blue (AquaCyp300). The refractive index (RI) signal profile (- -, dotted line) is shown throughout the elution volume. The calculated masses of 32 and 30 kDa are in good agreement to the exact masses of 31.8 kDa and 30.5 kDa. At the injected protein concentration of 1 mg/ml about 30% of AquaCyp300 is dimeric (peak at 63 kDa).
(TIF)

S3 Fig Assay of chaperone activity of AquaCyps. Influence of AquaCyp293 and AquaCyp300 on the aggregation of chemically denatured citrate synthase at 25°C. Denatured citrate synthase (30 μM in 6 M GdmCl, buffer) was diluted to a final concentration of 0.15 μM (monomer) in 100 mM Tris-HCl (pH 8.0), 1 mM EDTA, 30 mM GdmCl, 50 mM NaCl, and 0.1 mM DTE. Light scattering at 360 nm was monitored in the absence of a PPIase (○) and in the presence of 3.0 μM AquaCyp293 (□), of 3.0 μM AquaCyp300 (■) and, as a positive control, in the presence of 3.0 μM SlyD* (●).
(TIF)

S4 Fig Superposition of the AquaCyp293 and AquaCyp300 molecules in the crystallographic asymmetric unit and comparison to hCyp18 and EcCypB. Superposition of the two molecules of AquaCyp293 (A) and the six molecules of AquaCyp300 (B) in the crystal structure. The molecules in the asymmetric unit superimpose very well (rmsd <0.3 Å), suggesting low flexibility. Superimposition of AquaCyp293 (light gray; C,E) and AquaCyp300 (dark grey, D, F) to EcCypB (PDB ID: 2LOP; magenta) and hCyp18 (PDB ID: 2CPL; magenta).
(TIF)

S5 Fig Evolutionary relationship amongst cyclophilins of known structure. The phylogenetic tree was constructed by the neighbor-joining method based on cyclophilin structures deposited in the Protein Data Bank [81] as of January 1^st^, 2015. AquaCyp293 and AquaCyp300 cluster together in a separated sub tree.
(TIF)

S6 Fig Analysis of crystallographic temperature factors in the AquaCyp300 crystal structure. The crystallographic temperature (or B) factor is shown in a color-gradient for AquaCyp300 protomer2 from blue (low) to red (high). The active site of AquaCyp300 protomer1 is shown in yellow.
(TIF)

S7 Fig Distribution of hydrophobic and charged amino acids on AquaCyp293 and AquaCyp300. Surface representation of AquaCyp293 (A) and AquaCyp300 (B) in the same view and colored as in Fig 4A and 4B, respectively. (C,D) Surface representation of AquaCyp293 (C) and AquaCyp300 (D) color coded ranging from hydrophobic (green) to hydrophilic (grey) according to the normalized consensus hydrophobicity scale of the exposed residues [82], in the same orientation as in S6A and S6B Fig, repsectively. AquaCyp300 residues involved in dimerization (e.g. the C-terminal dimerization hairpin) are enriched in hydrophobic residues (E,F) Surface representation of of AquaCyp293 (E) and AquaCyp300 (F) color-coded ranging from negative charged (red) to positive charged (blue). The PPIase active site is indicated as ellipse.
(TIF)

## Abbreviations

PPIase: peptidyl-prolyl cis-trans isomerase
SEC-MALS: size exclusion chromatography coupled to multiangle light scattering
Abz: aminobenzoyl
pNA: para-nitroanilide
RCM-T1: reduced and carboxymethylated RNase T1
FKBP: FK506 binding protein
CS: citratesynthase
GdmCl: guanidinium chloride
