# Supplementary figures and images for: Structural and Functional Characterization of a Novel Family of Cyclophilins, the AquaCyps
Source: PLoS One. 2016 Jun 8;11(6):e0157070. doi: 10.1371/journal.pone.0157070 (PMC4898713; doi:10.1371/journal.pone.0157070)

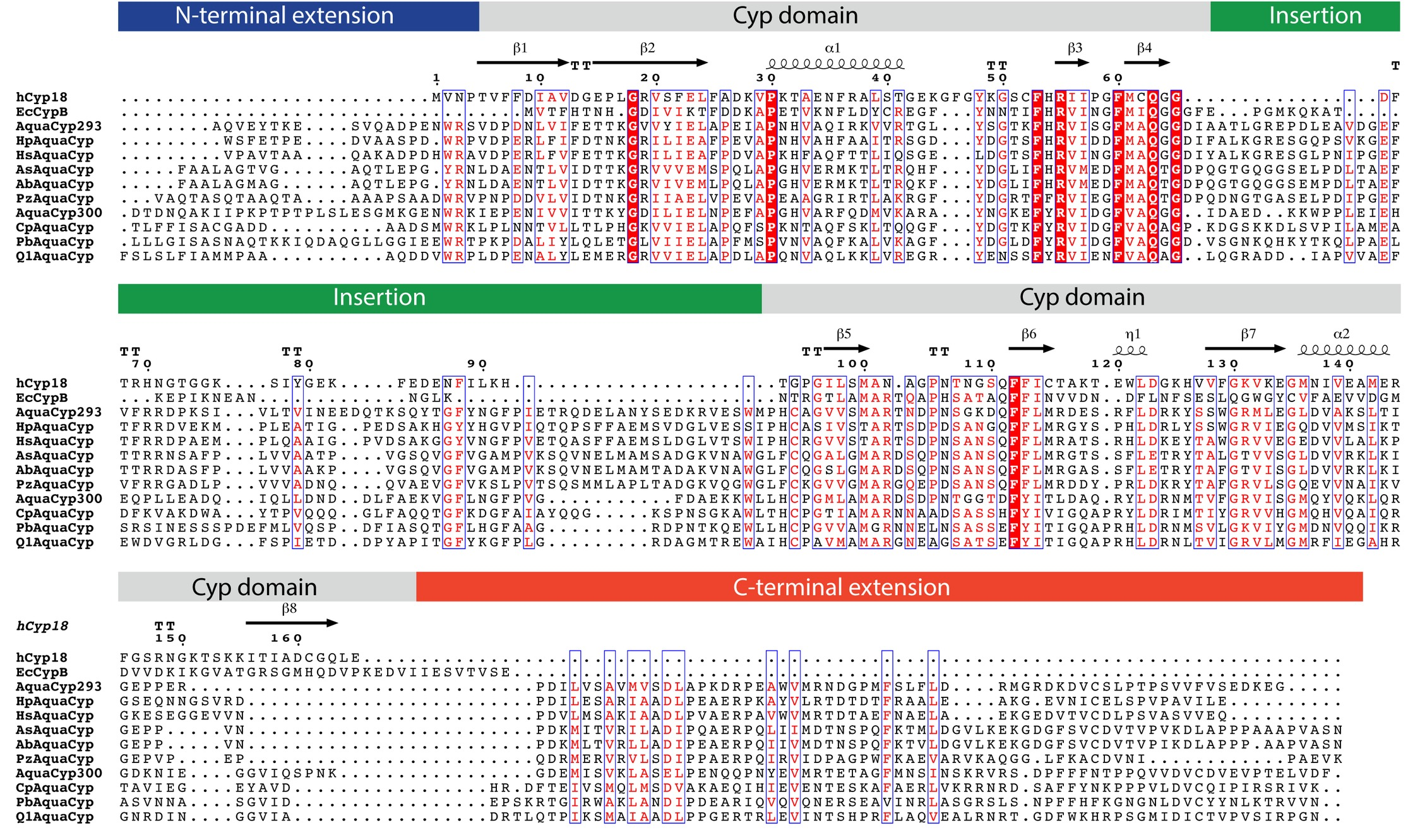

Supplement: S1 Fig — Multiple sequence alignment to analyze the sequence and secondary structure conservation of PrsA. Highly conserved residues are red (>70% conservation) or white in red boxes (100% conservation). On top, the cyclophilin domain are shown in grey, the additonal N-terminal-, insertion, and C-terminal structural elements are coloroured in blue, green and red, respectively. The secondary structure of Homo sapiens hCyp18 is shown on top of the protein sequence. Sequences of representative AquaCyp proteins were retrieved from the UniProt database [78] and aligned using MULTALIN [79]. The final figure was generated using the ESPript server [80]. Species abbreviations and UniProt accession numbers are AquaCyp293, Hirschia baltica (C6XJ17); HpAquaCyp, Hyphomonas polymorpha PS728 (A0A062V843); HsAquaCyp, Hyphomonas johnsonii MHS-2 (A0A059F982); AsAquaCyp, Asticcacaulis sp. AC460 (V4PTX4); AbAquaCyp, Asticcacaulis biprosthecum C19 (F4QR87); PzAquaCyp, Phenylobacterium zucineum (strain HLK1) (B4R9P8); AquaCyp300, Hirschia baltica (C6XII3); CpAquaCyp, Colwellia psychrerythraea (Vibrio psychroerythus) (A0A099L3T0); PpAquaCyp, Paraglaciecola polaris LMG 21857 (K6ZRP5); Q1AquaCyp, alpha proteobacterium Q-1 (A0A061QDA5). (TIF) [file pone.0157070.s001.tif]

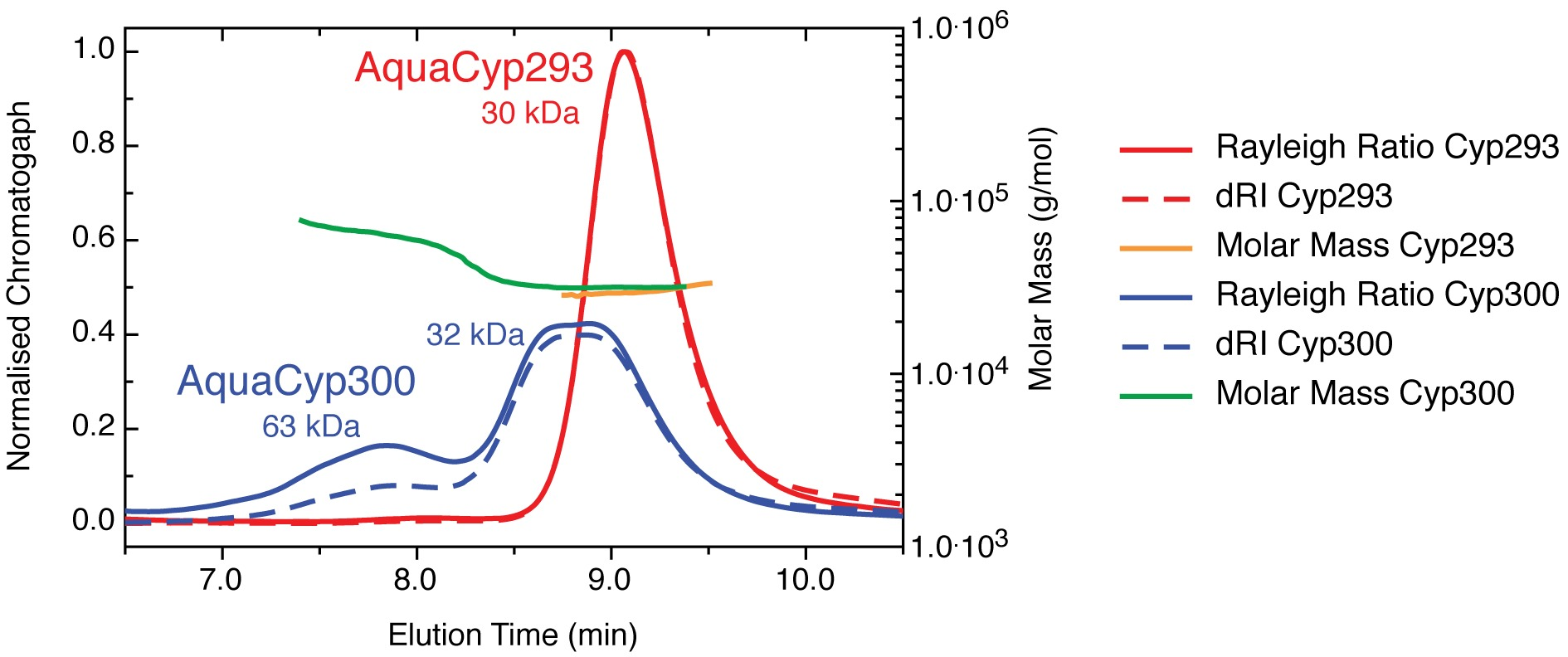

Supplement: S2 Fig — The chromatograms are shown for AquaCyp293 (red) and AquaCyp300 (blue). The molecular mass was calculated throughout the eluting peaks and is indicated in red (AquaCyp293) and blue (AquaCyp300). The refractive index (RI) signal profile (- -, dotted line) is shown throughout the elution volume. The calculated masses of 32 and 30 kDa are in good agreement to the exact masses of 31.8 kDa and 30.5 kDa. At the injected protein concentration of 1 mg/ml about 30% of AquaCyp300 is dimeric (peak at 63 kDa). (TIF) [file pone.0157070.s002.tif]

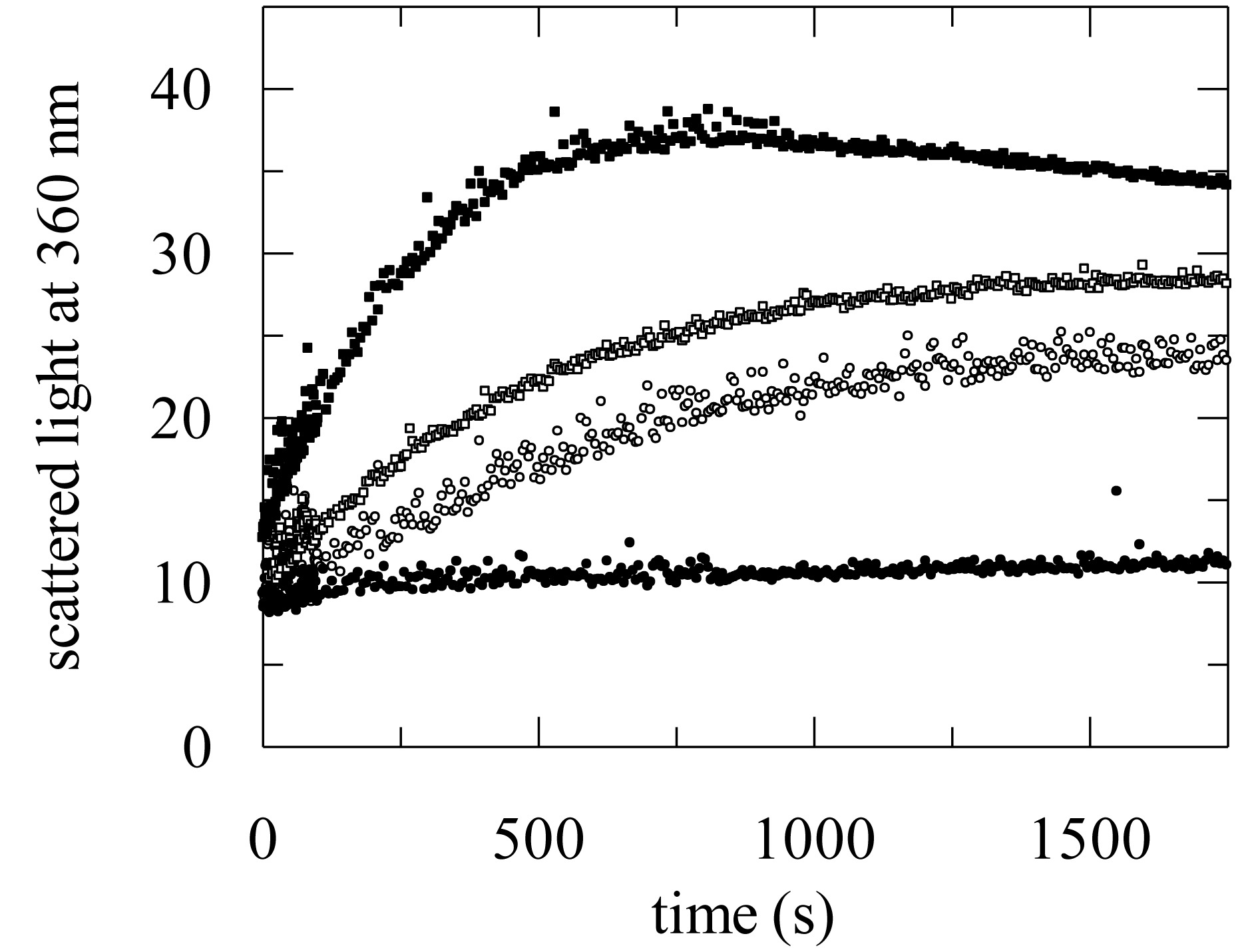

Supplement: S3 Fig — Influence of AquaCyp293 and AquaCyp300 on the aggregation of chemically denatured citrate synthase at 25°C. Denatured citrate synthase (30 μM in 6 M GdmCl, buffer) was diluted to a final concentration of 0.15 μM (monomer) in 100 mM Tris-HCl (pH 8.0), 1 mM EDTA, 30 mM GdmCl, 50 mM NaCl, and 0.1 mM DTE. Light scattering at 360 nm was monitored in the absence of a PPIase (○) and in the presence of 3.0 μM AquaCyp293 (□), of 3.0 μM AquaCyp300 (■) and, as a positive control, in the presence of 3.0 μM SlyD* (●). (TIF) [file pone.0157070.s003.tif]

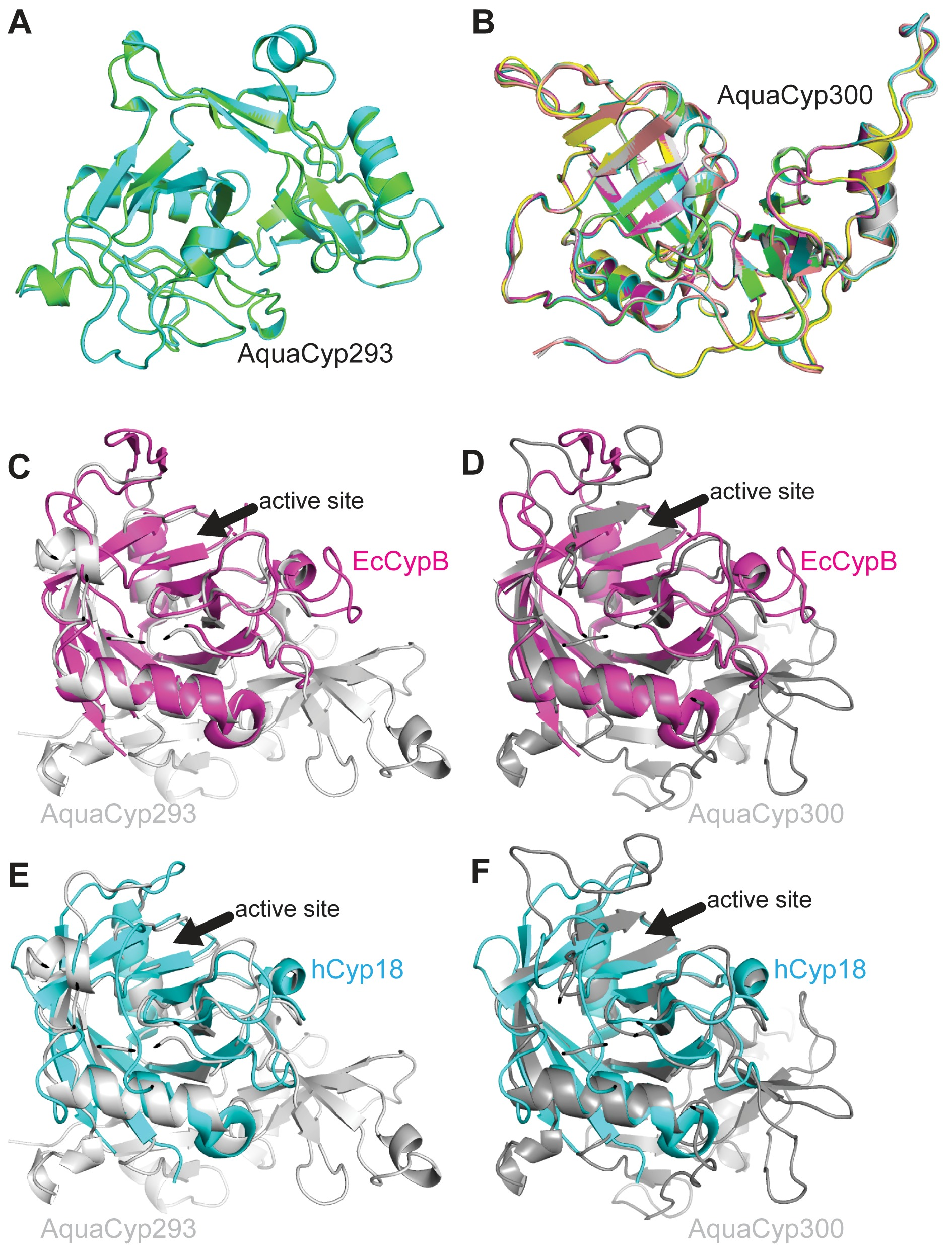

Supplement: S4 Fig — Superposition of the two molecules of AquaCyp293 (A) and the six molecules of AquaCyp300 (B) in the crystal structure. The molecules in the asymmetric unit superimpose very well (rmsd <0.3 Å), suggesting low flexibility. Superimposition of AquaCyp293 (light gray; C,E) and AquaCyp300 (dark grey, D, F) to EcCypB (PDB ID: 2LOP; magenta) and hCyp18 (PDB ID: 2CPL; magenta). (TIF) [file pone.0157070.s004.tif]

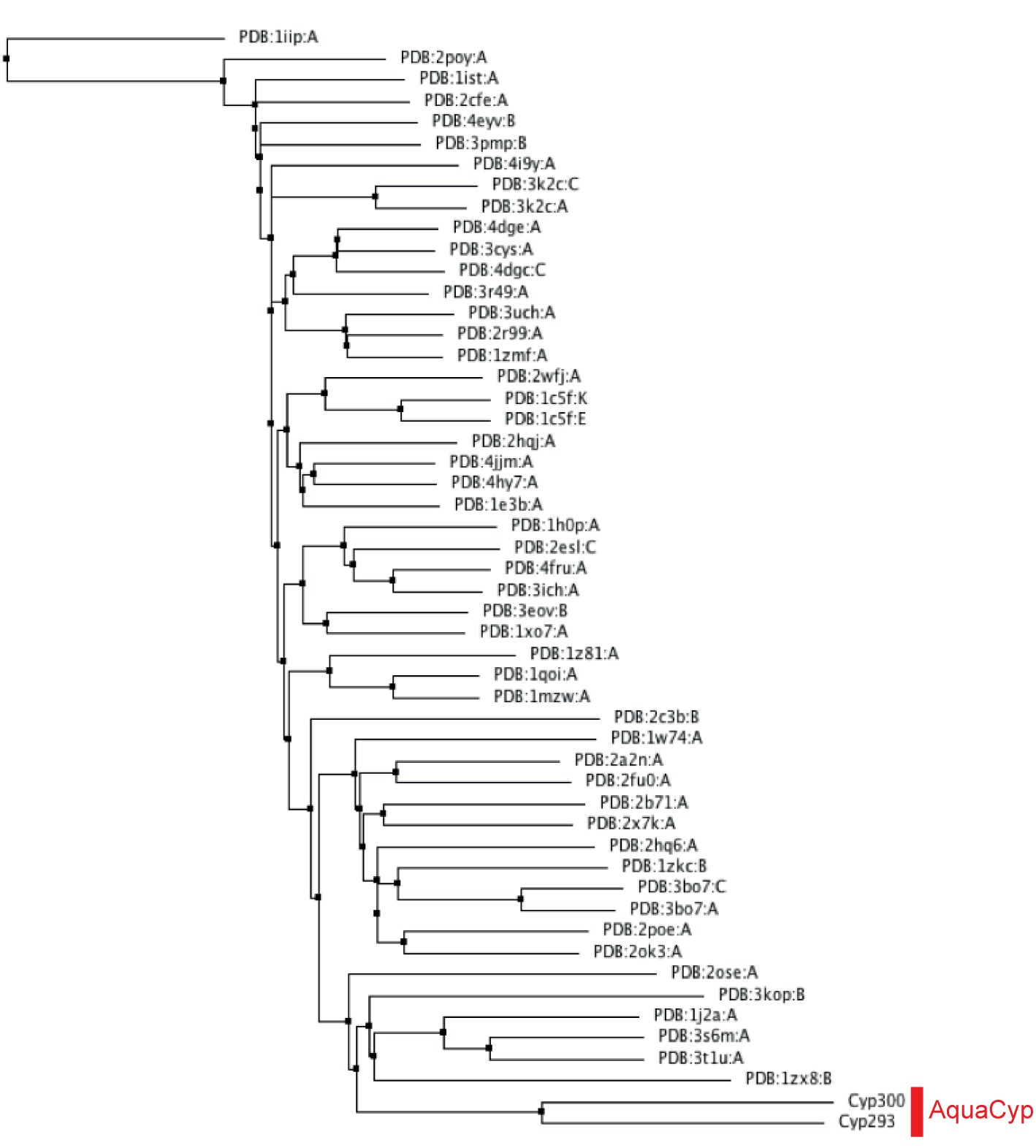

Supplement: S5 Fig — The phylogenetic tree was constructed by the neighbor-joining method based on cyclophilin structures deposited in the Protein Data Bank [81] as of January 1st, 2015. AquaCyp293 and AquaCyp300 cluster together in a separated sub tree. (TIF) [file pone.0157070.s005.tif]

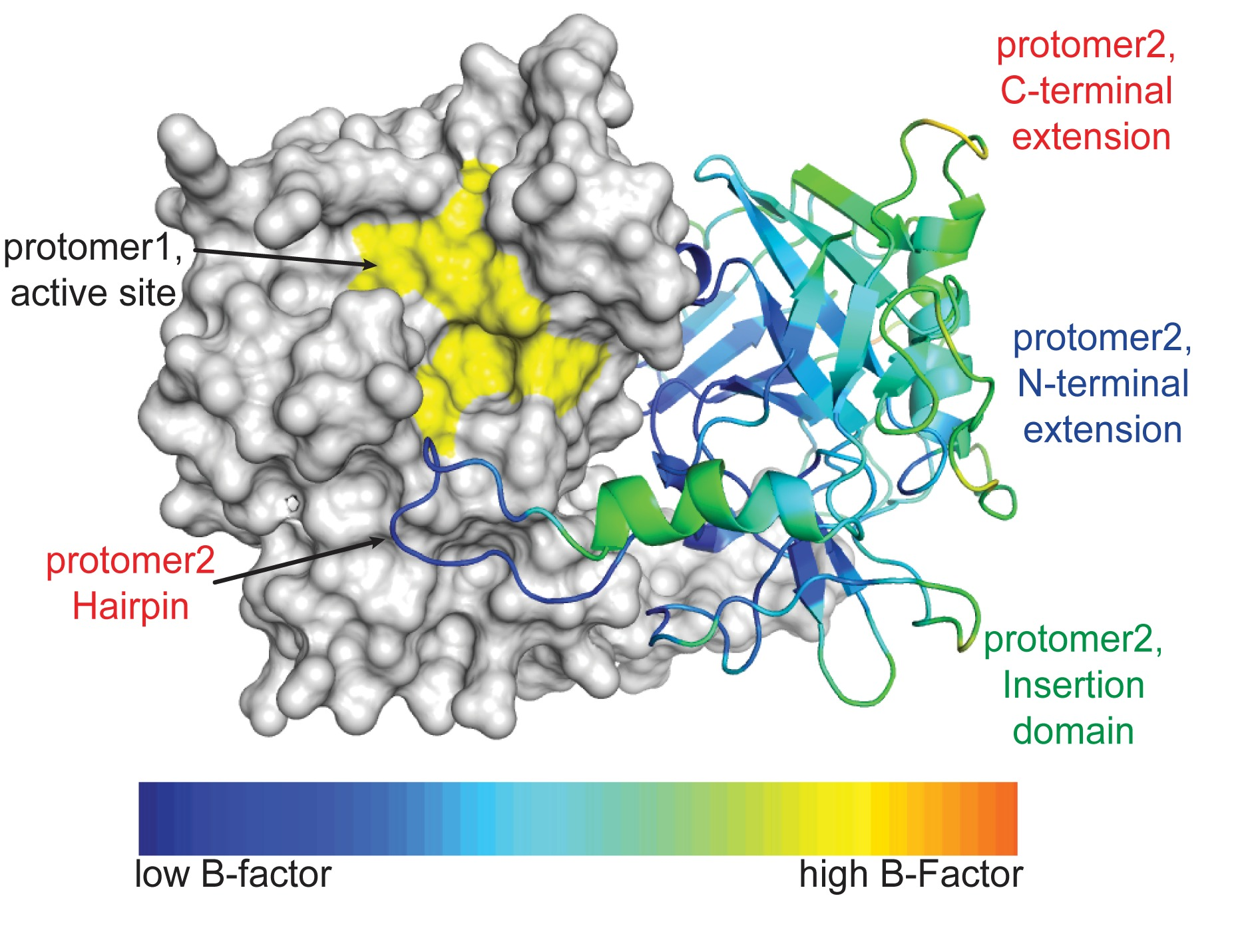

Supplement: S6 Fig — The crystallographic temperature (or B) factor is shown in a color-gradient for AquaCyp300 protomer2 from blue (low) to red (high). The active site of AquaCyp300 protomer1 is shown in yellow. (TIF) [file pone.0157070.s006.tif]

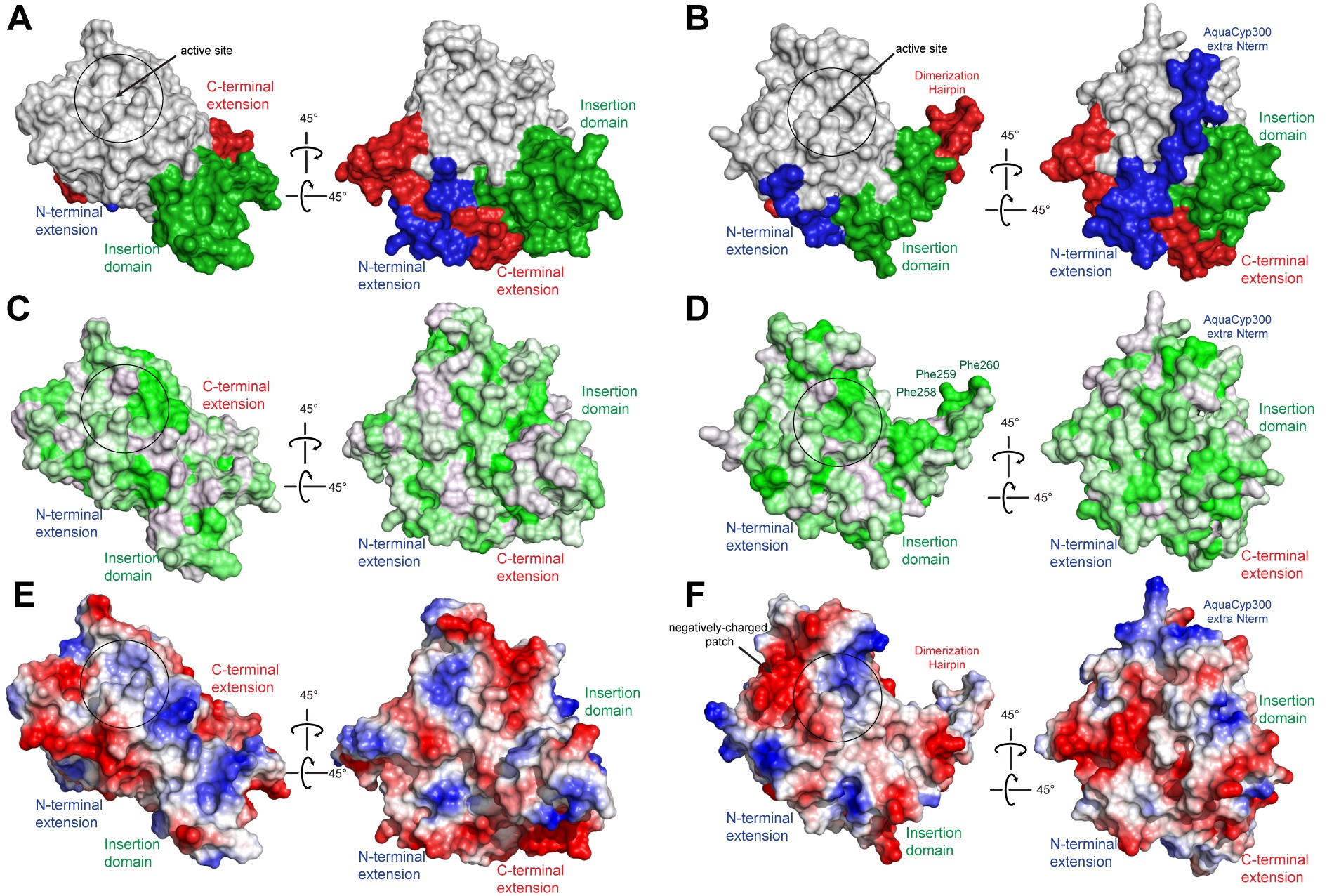

Supplement: S7 Fig — Surface representation of AquaCyp293 (A) and AquaCyp300 (B) in the same view and colored as in Fig 4A and 4B, respectively. (C,D) Surface representation of AquaCyp293 (C) and AquaCyp300 (D) color coded ranging from hydrophobic (green) to hydrophilic (grey) according to the normalized consensus hydrophobicity scale of the exposed residues [82], in the same orientation as in S6A and S6B Fig, repsectively. AquaCyp300 residues involved in dimerization (e.g. the C-terminal dimerization hairpin) are enriched in hydrophobic residues (E,F) Surface representation of of AquaCyp293 (E) and AquaCyp300 (F) color-coded ranging from negative charged (red) to positive charged (blue). The PPIase active site is indicated as ellipse. (TIF) [file pone.0157070.s007.tif]
